# Immediate chest radiograph interpretation by radiographers improves patient safety related to nasogastric feeding tube placement in children

**DOI:** 10.1007/s00247-021-05032-9

**Published:** 2021-03-10

**Authors:** Emily Keyte, Gillian Roe, Annmarie Jeanes, Jeannette K. Kraft

**Affiliations:** 1 Clarendon Wing Radiology Department, Leeds Children’s Hospital at the Leeds General Infirmary, Leeds, LS2 9NS UK; 2 Department of Radiology, St James’s University Hospital, Leeds, UK

**Keywords:** Children, Nasogastric tube, Patient safety, Paediatric radiologist, Radiography, Role expansion

## Abstract

**Background:**

Despite the publication of a national patient safety alert in 2016, inadvertent feeding through misplaced nasogastric tubes continues to occur, either through failure to review the radiograph, misinterpretation of it, or failure to communicate the results.

**Objective:**

The objectives were to determine whether training in a new pathway introduced to avoid these “never events” was followed and whether radiographer comments and prompt communication of results could reduce risk and improve patient safety in relation to nasogastric tube placement in children.

**Materials and methods:**

Following radiographer training in interpretation of nasogastric tube position and use of a commenting proforma and communication pathway, we reviewed all radiographs obtained to check nasogastric tubes performed over a 13-month period in children 0–16 years of age. Then we assessed accuracy of the radiographer comments, adherence to the pathway, and any practice change in children with misplaced nasogastric tubes.

**Results:**

We reviewed 282 nasogastric tube check radiographs. For 262 radiographs (92.9%) the pathway was followed correctly. Of the total 282 radiographs, 240 (85%) were immediately reported using the standardised commenting proforma, and 235 radiographer comments were affirmed by the radiologist (97% accuracy, confidence interval 0.95–0.99). Of the immediately reported radiographs, 213 (88.8%) nasogastric tubes were considered to be safe for use. Four (1.7%) of the immediately reported nasogastric tubes were misplaced in a bronchus, and the report communicated to the clinical team resulted in removal or re-siting of the tubes.

**Conclusion:**

Nasogastric tube check radiographs in children can be reported accurately by radiographers trained in their interpretation and the results promptly communicated to clinical staff, improving safety in relation to nasogastric tube placement in children.

## Introduction

Nasogastric tubes are commonly placed at a child’s bedside. Misplaced tubes compromise patient safety, with a risk of serious and potentially fatal complications. In 2016, the United Kingdom (UK) National Health Service (NHS) Improvement issued a safety alert titled “Nasogastric tube misplacement: continuing risk of death and severe harm” and a detailed resource set for hospital trust boards or equivalents with a list of actions to be implemented by April 2017 [1, 2]. The introduction of fluid or medication into the lungs or pleural space through a misplaced nasogastric tube was considered entirely avoidable and was officially listed as a “never event” [3].

The only accepted bedside method to confirm safe nasogastric tube placement prior to use is measurement of the pH of the aspirate obtained from the tube. A pH between 1 and 5.5 is considered safe to commence feeding [1, 2]. A properly obtained and reported chest radiograph is recommended in cases where no aspirate can be obtained or the pH indicator has failed to confirm a safe position of the tube. Radiography remains the gold standard for determining exact nasogastric tube tip location [4–6].

It is the responsibility of NHS Trusts and organisations providing NHS care that staff interpreting radiographs for nasogastric tube position receive competency-based training [2, 7]. However, in a review of 45 incidents related to radiograph interpretation in assessing nasogastric tube placement, none of the doctors involved had undergone competency-based training prior to the incident occurring [2].

It is the responsibility of the radiologist to report the nasogastric tube check radiograph, to document the position of the tube and tip. However, because of the increased complexity and demand for imaging, in parallel with a chronic shortage of radiologists in the UK, an immediate radiologist report is seldom available [8, 9].

Radiographer reporting comments have been used to assess and document the position of nasogastric tubes on radiographs in adults at our institution, a large tertiary hospital trust, since 2013. This has been shown to be a safe and extremely effective way to increase patient safety [10]. In 2018 this pathway was adapted and introduced for paediatric patients (0–16 years).

## Materials and methods

### The paediatric pathway

At the time of requesting the nasogastric tube check radiograph, the referrer is asked to provide the pH of the nasogastric aspirate to ensure that the aspiration test has been completed as a first-line investigation. The referrer is subsequently asked to confirm whether the child has had recent surgery for oesophageal atresia or a known hiatus hernia. The rationale for this is that the course of the nasogastric tube and position of the tip cannot be standardised in these cases. These examinations are classified “complex examinations” and are reported by a radiologist. Criteria are listed in Table 1.

**Table 1:** Standardised provisional report issued for a nasogastric tube check radiograph identified as a complex examination during the requesting process

Complex examination determinants for nasogastric tube check radiograph	
Note: The decision to feed lies with the staff on the ward.	
• A radiographer comment on the nasogastric tube position has not been provided for this radiograph because “yes” was answered to at least one of following questions at the time of requesting:	
- Does this patient have a nasojejunal tube in situ as well as a nasogastric tube?	
- Does this patient have oesophageal atresia?	
- Does this patient have a hiatus hernia?	
- Is this patient have postoperative gastric pull?	
• If an immediate radiologic report is required, this can be obtained upon request to a radiologist	
• A final interpretation will be provided by a radiologist	

Radiographs are performed in the radiology department or at the bedside for children in intensive care units. A nasogastric tube check radiograph is taken to include the chest and upper abdomen. After the radiograph has been acquired, a specific “nasogastric tube sticker” is attached to the end of the tube by the radiographer to highlight that a nasogastric tube check radiograph has been obtained. The sticker includes the date and time the radiograph was taken and the statement: “*Unconfirmed nasogastric tube position. Check radiograph and report before feeding.”* The radiograph is immediately reviewed by a radiographer who is trained in commenting on these studies, and the radiographer then provides a comment. The comment follows a standard proforma adapted from previously published literature in adults [10, 11] (Table 2). The radiographer also records answers to four questions on the Radiology Information System (Table 2). If all questions are answered “yes” then the tube is deemed safe for feeding. The radiographer comment includes an additional free text line to highlight any supplementary findings. Before using the nasogastric tube the ward doctor must review the radiographer comment and assess the radiograph. Once the nasogastric tube is considered safe to use, the nasogastric tube sticker can be removed.

**Table 2:** Standardised radiographer commenting proforma issued for a nasogastric tube check radiograph in children

Standardised radiographer comments for nasogastric tube check radiograph	
Notes: This is a provisional report focused on nasogastric tube position and has been issued by a radiographer. A final interpretation will be provided by a radiologist. The decision to feed lies with the staff on the ward.	
• Aspirate details: [*Details from request*]	
• The tube follows the line of the oesophagus: [*YES/NO*]	
• The tube bisects the carina: [*YES/NO*]	
• The tip passes below the diaphragm: [*YES/NO*]	
• The tip passes below the diaphragm: [*YES/NO*]	
• The crossing point is in the midline: [*YES/NO*]	
• Has the ward been contacted: [*YES/NO*]	
• Name of ward staff contacted (if applicable): [*Name of ward staff*]	
• Name of radiographer completing auto report: [*Name of reporting radiographer*]	
• Additional comments:	

If any questions are answered “no” then the tube is considered unsafe for use. This is documented and communicated directly with the ward. In contrast to adult practice, radiographers do not remove misplaced nasogastric tubes in children; the decision to remove the tube lies with the ward staff.

All paediatric nasogastric tube check radiographs are to be highlighted and priority reported by a radiologist within 24 h.

### Radiographer training

All radiographers undergoing training for paediatric nasogastric tube check radiographs must have previously completed training for nasogastric tube position interpretation in adults. Training is undertaken as a 1-h face-to-face group training session. This includes radiographic anatomy of the thorax, nasogastric tube check radiographic imaging techniques in children, and an outline of the referral and communication pathway for misplaced nasogastric tubes in children. A correctly placed nasogastric tube should follow the line of the oesophagus, bisect the carina and cross the diaphragm in the midline. The tip of the tube must lie below the diaphragm (Table 2). Multiple examples of correctly and incorrectly placed tubes are discussed during training to specifically highlight anatomical variants, drains, pacing wires and devices that might be encountered in paediatric patients. Electronic learning material is also provided. This consists of referral pathways, the presentation used for training, and examples of correctly and incorrectly placed tubes.

After completing the training, radiographers complete a nasogastric tube check radiograph interpretation test comprising 10 radiographs. The requirements of the test are to correctly comment on the position of the nasogastric tube course and tip. The radiographer must achieve 100% accuracy to be authorised to comment.

Protocols, flow charts and posters are also provided within the children’s radiology department to remind staff of safe and unsafe tube positions.

### Radiographer commenting audit

After radiographers were trained, we undertook a retrospective audit over a 13-month period, between October 2018 and October 2019, in children 0–16 years of age who had a undergone a nasogastric tube check radiograph. All radiographer commenting questions were evaluated and compared to the radiologist report to determine adherence the new pathway, to assess whether the commenting questions were answered accurately and to identify areas of improvement. Research ethics committee approval was not required because this was an evaluation of a change in working practice. The audit was registered according to Trust policy.

## Results

During the audit, 282 nasogastric tube check radiographs were performed. For 262 radiographs (92.9%), the correct pathway was followed. These included provision of a radiographer comment (240 radiographs), complex examination report (17 radiographs) and primary report by a radiologist (5 radiographs).

For 20/282 examinations (7.1%), the correct procedure was not followed. Ten examinations had no radiographer comment. Ten examinations had an incorrect comment and were either reported as for the adult nasogastric tube check radiograph pathway or provided a complex examination comment. The reasons for not following the correct pathway included: agency staff, newly qualified staff not fully aware of the new procedures and an incorrect assumption that requests from the the accident and emergency department were excluded from the pathway. In three cases no reason was apparent.

Of the 240 nasogastric tube check radiographs with a radiographer comment, 235 comments were fully agreed by the reviewing radiologist (97.9% accuracy, confidence interval 0.95–0.99). All five discrepancies related to the length of nasogastric tube below the diaphragm, whereby the radiologist advised that the tip of the nasogastric tube should be further advanced. The radiographer comments were otherwise correctly answered (100% accuracy) with 100% sensitivity and 100% specificity for the radiographer comment.

Of the 240 nasogastric tube check radiographs, 213 (88.8%) nasogastric tubes were considered to be safe for use, 27 (11.3%) were considered unsafe for use. Eleven (4.6%) nasogastric tubes were too short, with the tip sited in the oesophagus and in need of advancing. Sixteen nasogastric tubes needed removing, with 2 (0.8%) sited in the right main bronchus, 2 (0.8%) in the left main bronchus and 12 (5%) coiled within the oesophagus or pharynx.

## Discussion

Radiographer reporting was first introduced in the United Kingdom in the early 1980s, with the “red dot” scheme, whereby a radiographic abnormality identified by the radiographer was indicated by a red dot [12, 13]. Subsequently, the red dot scheme evolved into radiographer commenting, where a radiographer would also describe the appearance and location of the abnormality identified on a radiograph [12, 14]. Several studies have since demonstrated that reports from reporting radiographers who are trained to assess accident and emergency radiographs are as accurate as reports provided by radiologists [12, 15, 16].

Radiographer nasogastric tube commenting was introduced in our children’s hospital in 2018 after a child on the paediatric intensive care unit who had undergone complex cardiac surgery had a misplaced nasogastric tube that went unrecognised on three successive radiographs over a 24-h period. Unless a radiologist is contacted specifically to assess a radiograph, a formal radiologist report is not usually available immediately after tube placement, particularly when tube insertion takes place overnight or during the weekend. To our knowledge this is the case in many UK hospitals.

The experience of junior clinical doctors in interpreting nasogastric tube check radiographs can vary considerably. In 2015, Lee et al. [17] reported that it is not easy for paediatric residents to confirm the position of feeding tubes in neonatal radiographs and concluded that teaching or second opinions from radiologists or neonatal intensive care experts are needed to minimise complications. NHS Improvement recommended that all staff assessing radiographs for nasogastric tube position undergo competency-based training to assess appropriate nasogastric tube position and recognise malposition [2].

Our radiographer training involves a systematic approach for the assessment of chest radiographs of infants and children with nasogastric tubes in situ. The radiographer comment consists of a description of the course of the nasogastric tube from a trained health care practitioner who regularly assesses these radiographs. The key objective is to correctly and promptly identify all misplaced nasogastric tubes and communicate this directly to the ward staff so that removal or repositioning of the tube can be arranged.

Radiographer commenting has been shown to be both reproducible and accurate in our adult population [10]. The pathway and process for paediatrics, however, necessitated several changes from the adult nasogastric tube commenting system. For example, if a misplaced nasogastric tube is identified in an adult, it is removed by the radiographer whilst the patient is in the radiology department [10, 18]. Given the complexity of some children with nasogastric tubes at our institution, the potential distress caused, and the risks of removing an nasogastric tube unnecessarily, radiographer removal of nasogastric tubes in children was not considered appropriate. Our clinical practice in children, therefore, is for the commenting radiographer to highlight the malpositioned nasogastric tube to the responsible doctor, who will ultimately decide further management.

In our audit, all discrepancies between the radiographer comment and the radiologist report related to the length of the nasogastric tube below the diaphragm. In adult practice a well-positioned nasogastric tube is expected to reach at least 5 cm below the diaphragm. A specific measurement cannot be universally applied in children because of the wide age range and difference in size in children, especially neonates [10]. At our institution, we therefore expect a well-placed nasogastric tube tip to be sited well below the diaphragm to allow for side holes that can be present on some feeding tubes. This highlights the limitations of using a reporting proforma compared to a free text radiologist report. Since the audit, we have introduced an additional free text line to highlight when tube advancement should be considered before feeding or a tube is coiled in the oesophagus and might therefore not easily advance. This is often used by more experienced commenting radiographers.

Radiographers at our institution have greatly appreciated the opportunity for role extension through commenting on nasogastric tubes in both adult and paediatric practice and have played an important role in improving patient safety [18]. The commenting pathway was initially introduced to provide an immediate opinion on nasogastric tube position for radiographs requested under a specific nasogastric tube check radiograph code. Now that radiographers are trained and regularly comment on nasogastric tube positioning, they have since identified and highlighted multiple misplaced nasogastric tubes on chest radiographs performed for other reasons, further improving nasogastric patient safety and the quality of the service we provide at our institution.

## Conclusion

Nasogastric tube check radiographs in children can be reported accurately and promptly by radiographers trained in their positioning. The introduction of clear proformas and pathways ensures that misplaced nasogastric tubes are quickly identified and communicated to the clinical team, improving patient safety and the quality of care for children. We believe that radiographer nasogastric tube commenting could be introduced in paediatric radiology departments throughout the UK.
